# Device-Based Therapies for Refractory Angina

**DOI:** 10.3390/jcm14228013

**Published:** 2025-11-12

**Authors:** Andrea Caffè, Rocco A. Montone

**Affiliations:** 1 Department of Cardiovascular and Pulmonary Sciences, Catholic University of the Sacred Heart, 00168 Rome, Italy; 2 Department of Cardiovascular Medicine, Fondazione Policlinico Universitario A. Gemelli IRCCS, 00168 Rome, Italy

**Keywords:** refractory angina, stable angina, Coronary Sinus Reducer, device-based therapy, chronic coronary syndromes, ischemia with non-obstructive coronary arteries (INOCA), myocardial perfusion imaging, spinal cord stimulation, enhanced external counter-pulsation, extracorporeal shockwave myocardial revascularization

## Abstract

A substantial proportion of patients with ischemic heart disease continue to experience recurrent or persistent angina despite optimized medical therapy and prior revascularization, highlighting the need for novel and effective treatment strategies. Device-based therapies have emerged as promising options to address this unmet clinical need. Among these, the Coronary Sinus Reducer™ (CSR) has gained particular attention, supported by randomized trials and registries demonstrating improvements in angina symptoms and quality of life, with a favorable safety profile. However, a disconnect between symptomatic relief and objective measures of myocardial perfusion has been noted, suggesting that the underlying mechanisms remain incompletely understood. Beyond the CSR, other device-based approaches—such as enhanced external counterpulsation, neuromodulation, and shockwave therapy—are either approved or under investigation. This review explores the current landscape of device-based therapies for angina, focusing on the evidence supporting the CSR and other device-based interventions. We discuss pathophysiological mechanisms, clinical outcomes, and future research directions aimed at optimizing therapeutic efficacy. Integrating patient-reported outcomes with physiological and imaging assessments will be essential to refine indications and improve long-term results. Device-based therapies represent a developing frontier in angina management, with the potential to improve outcomes in patients with persistent symptoms despite optimal medical and interventional therapy.

## 1. Introduction: Unmet Clinical Needs in Refractory Angina

Angina pectoris remains a prevalent and clinically significant manifestation of ischemic heart disease (IHD), exerting a substantial impact on patients’ quality of life (QoL) and placing a considerable burden on healthcare systems worldwide [1]. Both obstructive coronary artery disease (CAD) and ischemia with non-obstructive coronary arteries (INOCA) are recognized as mechanisms underlying angina, reflecting the heterogeneous pathophysiology of this condition [2]. Contemporary medical therapy remains the first-line approach, aiming to reduce myocardial oxygen demand and/or enhance coronary perfusion [3,4,5]. In obstructive CAD, when symptoms persist despite optimal medical therapy (OMT), myocardial revascularization by percutaneous coronary intervention (PCI) or coronary artery bypass grafting (CABG) represents the reference strategy [3,4,5]. Moreover, myocardial revascularization confers a prognostic advantage over medical therapy alone in selected subsets, including patients with left main disease, diabetes with multivessel CAD, or reduced left ventricular ejection fraction (LVEF) [4].

Despite substantial advances in both pharmacological and interventional strategies, an estimated 30–40% of patients with chronic stable angina continue to experience symptoms despite OMT and prior revascularization [6,7]. When objective evidence of myocardial ischemia is present, this condition is defined as refractory angina [8]. According to the most recent European Society of Cardiology (ESC) guidelines for chronic coronary syndromes, refractory angina is defined as the persistence of symptoms for more than three months in the presence of documented reversible ischaemia [4]. This definition encompasses a heterogeneous spectrum of patients who are not amenable to revascularization, including those with unsuitable coronary anatomy, those in whom the risk-benefit profile precludes intervention, and those with coronary functional abnormalities beyond obstructive CAD [8]. In fact, while refractory angina is most frequently associated with obstructive CAD, the latest ESC guidelines also recognize that it can arise in the context of angina with non-obstructive coronary arteries (ANOCA), in which additional investigations are recommended to identify the specific endotype [4,9,10,11,12].

Refractory angina carries a substantial clinical burden, affecting a significant proportion of patients with CAD [13]. Beyond symptom persistence, it significantly compromises QoL and limits daily functioning [13]. Prognostically, in the OPTIMIST registry of patients with refractory angina, 5-year mortality approached 20%, predominantly from cardiovascular causes [14]. Similarly, patients with ANOCA exhibit an increased risk of MACE and all-cause mortality compared with the general population [15]. These observations highlight the unmet clinical need for innovative strategies to alleviate angina symptoms when conventional options have been exhausted.

## 2. Device-Based Therapies for Refractory Angina

A limited number of device-based therapies have been developed for the management of refractory angina, either by enhancing myocardial perfusion or by modulating cardiac nociception [8]. Among these, the Coronary Sinus Reducer™ currently represents the only device-based therapy for chronic angina recommended by the 2024 ESC Guidelines (Class IIb, Level of Evidence B) [4]. While other modalities such as enhanced external counterpulsation (EECP), spinal cord stimulation (SCS), and extracorporeal shockwave myocardial revascularization (ESMR) have shown symptomatic benefit in selected studies, their clinical use remains limited [13].

### 2.1. Coronary Sinus Reducer^TM^

The Coronary Sinus Reducer™ (originally developed and marketed by Neovasc Inc., Richmond, BC, Canada, currently commercialized by Shockwave Medical Inc., Santa Clara, CA, USA) represents an extensively investigated device-based therapy for patients with refractory angina not amenable to percutaneous or surgical revascularization [16]. In 2018, the U.S. Food and Drug Administration (FDA) granted the Reducer™ a “Breakthrough Device” designation, which does not constitute market approval [17]. In Europe, the device has received CE marking and is clinically available for patients with refractory angina unsuitable for revascularization [16].

The Reducer™ is a balloon-expandable, hourglass-shaped stainless-steel mesh device designed to create a permanent focal narrowing within the lumen of the coronary sinus (CS), thereby generating a pressure gradient [13]. The anti-anginal and anti-ischemic effects of the Reducer™ are attributed to a controlled elevation of backward pressure in the coronary venous system, which induces modest dilation of subendocardial arterioles, and enhances perfusion of ischemic myocardial layers, as demonstrated in preclinical models where coronary sinus occlusion increased subendocardial blood flow and improved the endocardial-to-epicardial perfusion ratio [8,18]. By chronically increasing venous pressure, the Reducer™ may promote a redistribution of myocardial blood flow toward ischemic subendocardial regions, alleviating ischemia, and improving anginal symptoms [8,13,19]. Recent evidence suggests that the effectiveness of the Reducer™ is not limited to left coronary territories. Studies have reported clinical benefit also in patients with right coronary artery disease (RCA) or even chronic total occlusion of the RCA, suggesting that its hemodynamic and microvascular effects may extend beyond the anatomical drainage of the left coronary system [20,21].

Procedural considerations are critical to maximize safety and efficacy. Patients are typically pretreated with aspirin in combination with a P2Y12 inhibitor, and dual antiplatelet therapy is recommended for six months following Reducer™ implantation [22,23]. Following right jugular venous access, right atrial pressure is measured with a multipurpose diagnostic catheter. A mean pressure above 15 mmHg is considered a contraindication, as the hemodynamic effect of the Reducer™ is likely to be attenuated in this setting [23,24]. After confirming eligibility, the CS is engaged, with contrast injection to verify ostial access [23]. The Reducer™ is advanced over a supportive wire and deployed in the optimal landing zone—usually 1.5–3 cm distal to the ostium—by inflating the balloon at 4–6 atm for at least 60 s, with 10–20% oversizing to ensure anchoring. Careful balloon retrieval and final venography confirm correct positioning and absence of complications [23]. A few weeks after implantation, the spaces between the metal struts are covered by endothelialization, leading to narrowing of the lumen and the formation of a pressure gradient across the device [8].

Available evidence indicates that implantation of the Reducer™ is associated with a favorable safety profile, with severe procedural complications being uncommon [16,25]. The most relevant adverse events include CS dissection and perforation, typically related to guidewire migration into small tributaries or to device oversizing. In most cases, conservative management with protamine reversal and prolonged balloon tamponade is sufficient [26]. Device-related issues mainly consist of scaffold migration, usually occurring during balloon retrieval in the setting of inadequate anchoring, and entrapment of the guiding catheter at the device neck [23].

### 2.2. Spinal Cord Stimulation (SCS)

SCS is a neuromodulation therapy that has been shown to reduce angina frequency and nitrate use, while improving exercise tolerance and QoL in patients with refractory angina [27,28]. The proposed mechanisms include modulation of nociceptive transmission within the dorsal columns, enhanced release of inhibitory neuropeptides, and normalization of intrinsic cardiac neuronal activity. Together, these effects attenuate cardiac pain perception and may promote redistribution of coronary blood flow to ischemic regions [29]. A typical therapeutic regimen consists of three one-hour low-amplitude stimulations per day, with additional on-demand sessions during angina attacks [8].

However, most outcome data are derived from small open-label studies. The largest randomized trial, ESBY (Electrical Stimulation versus Coronary Artery Bypass Surgery), included 104 patients and demonstrated symptom relief in both groups. Although improvements were greater with CABG, SCS was associated with lower mortality and cerebrovascular morbidity [30].

Reported complication rates for SCS reach 30–40%, with adverse events being either hardware-related (lead migration, fracture, or device failure) or biological (infection, pain at the implant site, dural puncture headache, or, rarely, neurological injury) [8]. Moreover, peri-procedural interruption of antithrombotic therapy may raise specific concerns in patients with advanced CAD [8].

### 2.3. Extracorporeal Shockwave Myocardial Revascularization (ESMR)

ESMR involves the application of low-energy acoustic shockwaves to ischemic myocardial regions, delivered through an external generator under echocardiographic guidance for accurate targeting [31]. A typical treatment protocol includes nine sessions, each consisting of approximately 1000 impulses distributed across multiple ischemic sites. Reported contraindications are few and include poor acoustic windows and the presence of left ventricular thrombus [8]. Shockwave-induced mechanotransduction promotes vasodilation and stimulates angiogenesis and neovascularization through the activation of multiple signaling pathways [31].

Clinical evidence has reported improvements in angina symptoms, exercise tolerance, ischemic thresholds, and health-related QoL [32,33]. Some studies have also demonstrated reductions in ischemic burden and modest improvements in LVEF [34,35]. The therapy has been reported as well tolerated, with no major safety concerns [36].

A meta-analysis of 39 studies further supported the potential of ESMR, while highlighting the need for adequately powered trials to establish its efficacy and durability [36].

### 2.4. Enhanced External Counter-Pulsation (EECP)

EECP is a non-invasive therapy developed to improve coronary perfusion in patients with refractory angina. The system consists of three pairs of pneumatic cuffs applied to the lower limbs, which inflate sequentially to approximately 300 mmHg during early diastole and deflate before systole, synchronized with the electrocardiogram [8]. A full treatment course typically comprises 35 one-hour sessions delivered five times per week over seven weeks [13]. The hemodynamic effects of EECP include diastolic augmentation of arterial pressure and retrograde aortic blood flow, thereby enhancing coronary perfusion and reducing left ventricular afterload. On the venous side, EECP enhances right ventricular preload by increasing venous return [13]. Additionally, the increased coronary shear stress during therapy induces endothelial-dependent vasodilation, and may stimulate neovascularization [8,37].

The randomized MUST-EECP trial compared active therapy with sham counterpulsation in 139 patients with documented ischemia, demonstrating significant benefit in angina symptoms and delayed onset of ischemia with EECP [38]. These findings have been supported by subsequent studies, showing sustained symptomatic relief in many patients for up to two years [39].

EECP is generally well tolerated, with adverse events limited to mild, equipment-related complications [8]. The therapy is endorsed by the 2023 AHA/ACC guidelines for the management of patients with chronic coronary disease (Class IIb, Level of Evidence B) [40].

### 2.5. Outdated Approaches: Transmyocardial Laser Revascularization (TMLR), Percutaneous Myocardial Laser Revascularization

TMLR was developed to treat refractory angina by creating transmural channels in ischemic myocardium, with the aim of restoring perfusion, stimulating angiogenesis, and inducing myocardial sympathetic denervation [8,41]. Both surgical (epicardial) and percutaneous (endocardial) approaches were investigated. Open-label trials initially suggested symptomatic benefit, accompanied by substantial perioperative risks, including mortality rates of 3–5%, as well as higher incidence of heart failure, myocardial infarction, and thromboembolic events compared with medical therapy [8,42]. The only sham-controlled trial of percutaneous TMLR failed to demonstrate symptomatic benefit and raised safety concerns [43], while a meta-analysis concluded that the risks of TMLR outweigh any potential clinical benefits [44]. Consequently, neither surgical nor percutaneous TMLR is recommended by current guidelines [4,40].

## 3. Clinical Evidence for the Coronary Sinus Reducer™

The evidence supporting the Reducer™ is considered of moderate strength, derived from randomized controlled trials (RCTs) of modest sample size, meta-analyses, and real-world registries. Collectively, these data underpin the recommendations in the most recent ESC guidelines for the management of chronic coronary syndromes (2024), which assign the Reducer™ a Class IIb, Level of Evidence B indication for the management of refractory angina [4], while the 2023 AHA/ACC guidelines for the management of patients with chronic coronary disease do not specifically address the use of the Reducer™ [40].

Clinical evidence has evolved from small randomized proof-of-concept studies [45] to larger observational programs [16,25] and more recent placebo-controlled mechanistic trials [24]. In the COSIRA randomized trial, implantation of the Reducer™ in patients with refractory angina resulted in significantly greater improvements in angina class, assessed by the Canadian Cardiovascular Society (CCS) grading, and in QoL, measured by the Seattle Angina Questionnaire (SAQ), compared with a sham procedure [22]. Subsequent single-arm experiences confirmed high procedural success, consistent symptomatic improvement, and a favorable safety profile [46,47].

The RESOURCE registry provided a comprehensive real-world perspective on the use of the Coronary Sinus Reducer™ in refractory angina, reporting high procedural success (96.7%) and a low overall complication rate (5.7%) among 658 patients, with no intra- or periprocedural deaths or myocardial infarctions [25]. At a median follow-up of 17 months, 39.7% of patients achieved a ≥2-class improvement in CCS score and 76% improved by at least one class, indicating a meaningful and durable symptomatic benefit [25]. Consistent with these findings, the REDUCER-I program, which enrolled 400 patients with refractory angina, showed that 69.8% experienced at least a one-class CCS improvement, accompanied by significant gains in exercise tolerance [16]. However, this cohort included a notable proportion of patients below CCS class III (28%) and approximately one-third on fewer than three antianginal agents [16].

The recent randomized, double-blind ORBITA-COSMIC trial evaluated the Reducer™ in 50 patients with refractory angina, stable CAD, documented ischemia, and no further revascularization options [24]. Using quantitative adenosine-stress perfusion cardiac magnetic resonance (CMR) and daily symptom tracking via the ORBITA-app, the trial assessed both objective myocardial perfusion and patient-reported angina [24]. The device produced a significant reduction in daily angina episodes, evident from approximately 10 weeks post-procedure and sustained to six months, alongside improvements in SAQ angina frequency and MacNew QoL scores [24]. The Reducer™ did not significantly improve global myocardial blood flow on stress CMR compared with placebo, as the primary imaging endpoint was negative [24]. However, a significant improvement in blood flow was observed in the ischemic subendocardium (a prespecified secondary endpoint), which may account for the reduction in angina symptoms and the improvement in quality of life observed in patients treated with the device [24]. These symptomatic benefits occurred despite a background of intensive antianginal therapy, with patients taking a median of three agents, and were achieved with high blinding fidelity, minimal procedural complications, and absence of MACE [24].

Furthermore, a 2025 meta-analysis including 1565 patients from 20 studies showed that Reducer™ implantation was associated with substantial symptomatic and functional benefits, with 70% of patients achieving at least a one-class improvement in CCS class, consistent improvements in all domains of the SAQ, and significant gains in six-minute walk distance, alongside a very high implantation success rate (99%) and a low periprocedural complication rate (2%), supporting Reducer™ as a safe and effective option for patients with refractory angina [48]. Moreover, a 2020 health technology assessment indicates that Reducer™ implantation reduces angina-related healthcare resource use and costs [49].

Emerging evidence suggests that the Reducer™ may offer symptomatic and physiological benefits in patients with ANOCA. Insights into the effects of the Reducer™ on microvascular coronary physiology were provided by the INROAD study, which enrolled 24 patients with refractory angina and obstructive CAD who underwent device implantation [50]. Invasive coronary physiology assessment was performed in a patent, non-grafted coronary artery at baseline and four months after implantation [50]. The study reported a significant reduction in the index of microcirculatory resistance (IMR), with 71% of patients achieving ≥20% IMR decrease, accompanied by improvements in coronary flow reserve (CFR) and resistive reserve ratio. Clinically, 76% of patients experienced at least one-class improvement in CCS class [50]. Furthermore, a phase II study in patients with ANOCA demonstrated that Reducer™ implantation improved both CFR and acetylcholine-mediated coronary blood flow, alongside improvements in CCS class and SAQ [51]. Moreover, a randomized, sham-controlled crossover trial demonstrated that transient coronary sinus occlusion induces an acute reduction in microvascular resistance—reflected by a shorter mean transit time and lower distal coronary pressure, particularly during hyperemia—indicating improved coronary flow in patients with ANOCA [52].

Consistently, registry data indicate that patients with refractory symptoms and objective myocardial ischemia derive comparable symptomatic benefit from Reducer™ implantation irrespective of the presence of obstructive or non-obstructive CAD, further supporting its potential role across the refractory angina spectrum [53]. However, the nonrandomized study designs and small patient populations warrant cautious interpretation of the efficacy of Reducer™ in patients with refractory ANOCA.

## 4. Clinical Implications and Current Controversies

**Figure 1:**
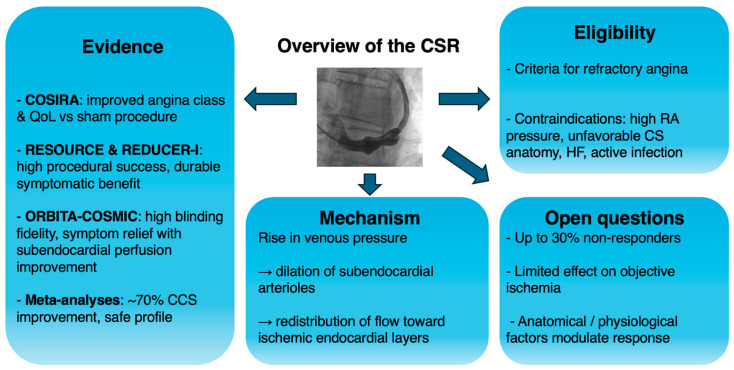
Overview of the Coronary Sinus Reducer™. Abbreviations: CCS: Canadian cardiovascular society; CS: coronary sinus; CSR: Coronary Sinus Reducer; HF: heart failure; QoL: quality of life; RA: right atrium.

Taken together, current evidence supports the Coronary Sinus Reducer™ as a promising anti-anginal option for carefully selected patients, but uncertainty persists regarding objective perfusion effects, responder phenotypes, and long-term outcomes [54] (Figure 1).

In this context, patient selection criteria include stable, refractory symptoms (CCS class III–IV) despite OMT, objective evidence of myocardial ischemia, and the absence of revascularization options for anatomical, technical, or clinical reasons [16,22,24,54].

The diagnosis of ischemia should be confirmed by functional imaging—such as stress CMR, single photon emission computed tomography (SPECT), or stress echocardiography—or invasive coronary physiological assessment, consistent with inclusion criteria used in randomized trials and multicenter registries [16,24,53]. Contraindications include unfavorable CS anatomy, right atrial pressure > 15 mmHg, presence of a biventricular pacing lead within the CS, decompensated advanced heart failure, severe valvular disease, CS thrombosis, active infection, or limited life expectancy due to non-cardiac comorbidities [22,23,24,25].

Despite the overall efficacy of the Reducer™, up to 30% of patients derive minimal or no benefit, underscoring the need for careful patient selection and a deep mechanistic understanding [22,55]. Symptomatic improvement may occur both in patients with non-revascularizable obstructive CAD and in those with ANOCA, provided that objective evidence of ischemia is documented [53]. Instead, other factors may modulate the effectiveness of Reducer™ implantation. Anatomical considerations include cardiac venous system heterogeneity, as patients with extensive alternative drainage pathways (e.g., Thebesian veins) may experience smaller CS pressure increases during implantation and only limited benefit [56]. Furthermore, large or unusually shaped CS segments may reduce effective pressure gradients, and incomplete device endothelialization may limit retrograde venous pressure [56]. Moreover, a larger rise in differential pressure between baseline right atrial pressure and CS systolic pressure during balloon occlusion of the CS has been suggested to predict clinical responsiveness to Reducer™ implantation [57]. Overall, these findings highlight the importance of integrating anatomical and physiological assessments to improve patient selection and maximize the likelihood of a durable response to Reducer™ therapy.

From a safety perspective, procedural risks such as cardiac tamponade or device embolization, although infrequent, have been reported, emphasizing the importance of eligibility assessment and procedural expertise [58].

The discrepancy between patient-reported angina symptoms and objectively measured myocardial ischemia represents a key focus in Coronary Sinus Reducer™ research. Assessment of myocardial ischemia and perfusion varied across studies, including exercise duration, evaluation of myocardial perfusion using either SPECT or positron emission tomography (PET), dobutamine stress echocardiography, and adenosine-stress perfusion CMR, with objective improvements in perfusion not consistently observed [22,24,45,55]. This heterogeneity has been addressed in a recent meta-analysis [58], which confirmed improvements in angina class and QoL but highlighted limited and inconsistent effects on global ischemia endpoints, especially in placebo-controlled settings.

Konigstein et al. reported in 23 patients that stress-induced myocardial function improved six months after Reducer™ implantation, with better wall motion score index (WMSI) and LVEF on dobutamine stress echocardiography, and reduced ischemic burden on thallium stress scans, while the results of the treadmill stress test did not reach statistical significance [45]. In the subsequent larger COSIRA randomized trial (including 104 patients), six months after Reducer™ implantation, there were non-significant improvements in total exercise duration, time to 1 mm ST-segment depression, and WMSI on dobutamine stress echocardiography, suggesting a limited impact on objective ischemia despite symptomatic benefit [22]. Furthermore, in the REDUCE study, treadmill exercise duration did not significantly improve after Reducer™ implantation, and imaging findings were heterogeneous: while myocardial scintigraphy showed a reduction in ischemia extent and dobutamine stress echocardiography demonstrated lower prevalence of inducible ischemia, no significant changes were observed in WMSI or LVEF [55]. More recently, ORBITA-COSMIC demonstrated a dissociation between ischemia assessment by perfusion CMR and clinical benefit in patients treated with the Reducer™ [24]. This discrepancy suggests that symptomatic relief may derive from mechanisms other than global perfusion enhancement, potentially including redistribution of subendocardial blood flow within ischemic territories [24].

A perfusion CMR study demonstrated that Reducer™ implantation reduced ischemic burden and restored the endocardial-to-epicardial perfusion ratio in previously ischemic segments, paralleled by improvements in left ventricular systolic function and strain indices [59]. Complementing these observations, a pilot CMR perfusion study showed that, although global myocardial blood flow and perfusion reserve did not change significantly, preferential perfusion improvements occurred in the most ischemic territories on segmental analysis, with redistribution toward the endocardium [60]. These results support the hypothesis that Reducer™ primarily exerts its benefit through regional perfusion redistribution rather than global flow augmentation (Table 1).

## 5. Future Directions

Ongoing randomized trials are expected to expand the evidence base for the Coronary Sinus Reducer™. The COSIRA-2 trial (Efficacy of the COronary SInus Reducer in Patients with Refractory Angina II; NCT05102019), currently enrolling, is powered to evaluate changes in total exercise duration on treadmill testing in patients with refractory angina and will also include assessments of microvascular function [61]. In parallel, the COSIMA (Coronary Sinus Reducer for the Treatment of Refractory Microvascular Angina) trial (NCT04606459) is investigating the role of the device in patients with microvascular angina, addressing a population in whom clinical benefit remains uncertain [62]. Another study specifically targets patients with INOCA: the REMEDY-PILOT (Reducing Microvascular Dysfunction in Patients with Angina, Ischaemia and Unobstructed Coronary Arteries—A Pilot Study) study (NCT05492110), designed to evaluate feasibility and preliminary efficacy in patients with ANOCA, and its mechanistic REMEDY-MECH substudy, which aims to characterize physiological changes underlying symptomatic response [63].

Collectively, these trials are expected to clarify not only the magnitude of clinical benefit from Reducer™ implantation, but also the underlying mechanisms and optimal patient selection across both obstructive and non-obstructive CAD phenotypes.

## 6. Conclusions

Refractory angina remains a clinically challenging condition resulting from heterogeneous pathophysiological mechanisms, including obstructive and non-obstructive CAD. Despite advances in pharmacological therapy and revascularization techniques, a substantial proportion of patients continue to experience persistent and disabling symptoms, underscoring the unmet clinical need for alternative interventions [13]. Device-based therapies—most notably the Coronary Sinus Reducer™—have demonstrated consistent improvements in angina class, exercise capacity, and patient-reported QoL in both randomized trials and real-world registries [54]. Procedural safety is generally favorable, although careful patient selection remains critical, and objective measures of global myocardial perfusion show variable results [58]. Ongoing studies, including COSIRA-2, COSIMA, and REMEDY-PILOT, are expected to elucidate the mechanistic effects of the device, define responder phenotypes, and refine selection criteria across both obstructive and non-obstructive CAD populations [61,62,63]. Collectively, these findings highlight the importance of integrating anatomical, physiological, and patient-reported assessments to guide evidence-based application of device-based therapies in refractory angina.

## Figures and Tables

**Table 1:** Studies assessing ischemia-related endpoints after Coronary Sinus Reducer™ implantation.

Study/Author	Design/Population	Number of Patients/Follow-Up	Ischemia Endpoint	Main Results
Konigstein et al. (2014) [45]	Prospective, single-center; RA patients	23 patients/6 months	Dobutamine stress echo (WMSI, LVEF), thallium stress scan, treadmill	Improved WMSI and LVEF; reduced ischemic burden on thallium; treadmill test not significant.
COSIRA (2015) [22]	Randomized, double-blind sham-controlled; RA patients	104 patients/6 months	Treadmill exercise duration, time to 1 mm ST depression, WMSI (dobutamine)	Significant angina and QoL benefit vs. sham; no consistent improvement in ischemia endpoints.
REDUCE (2018) [55]	Multicenter, prospective registry; RA patients	85 patients (imaging subset)/6–12 months	SPECT, dobutamine stress echo, treadmill, 6-MWT	Reduced ischemia extent on SPECT; lower prevalence of inducible ischemia on echo; WMSI, LVEF, and exercise time not significant.
ORBITA-COSMIC (2024) [24]	Randomized, double-blind; RA patients	50 patients/6 months	Quantitative adenosine-stress perfusion CMR (global & segmental MBF, endo/epi ratio)	Reduced daily angina, improved QoL; no global perfusion gain; evidence of subendocardial redistribution.
INROAD (2024) [50]	Multicenter, prospective; post-revascularization RA patients	24 patients/4 months	Invasive microvascular testing: IMR, CFR, resistive reserve ratio	Significant IMR reduction (71% ≥ 20%); CFR improvement; supports effect on microcirculation.
Tryon et al. (2024) [51]	Phase II trial, ANOCA patients with coronary microvascular dysfunction	30 patients/4 months	Microvascular function (CFR, CBF in response to acetylcholine)	Significant increase in both CFR and CBF in response to acetylcholine
Ullrich et al. (2023) [52]	Sham-controlled, crossover, randomized; refractory ANOCA patients with IMR > 25	20 patients	Aortic and distal coronary pressure, coronary sinus pressure, right atrial pressure, mean transit time	Significantly reduced IMR and improved coronary blood flow during acute ballon inflation in CS
Palmisano et al. (2021) [59]	Multiparametric CMR with feature-tracking & mapping; RA patients	28 patients (20 analyzed)/4 months	Stress perfusion CMR (MPR), ischemic burden, strain, T1/ECV mapping	Reduced ischemic burden; restored endocardial/epicardial ratio; improved LVEF and strain; no change in T1/ECV.
Cheng et al. (2025) [60]	Pilot quantitative perfusion CMR; RA patients	16 patients/3–4 months	Quantitative perfusion CMR: global & segmental MBF/MPR, endo/epi ratio	No global MBF/MPR change; segmental improvements in most ischemic regions with endocardial redistribution.

Abbreviations: 6-MWT: 6 min walking test; ANOCA: angina with non-obstructive coronary arteries; CBF: coronary blood flow; CFR: coronary flow reserve; CMR: cardiac magnetic resonance; CS: coronary sinus; ECV: extracellular volume; IMR: index of microcirculatory resistance; LVEF: left ventricular ejection fraction; MBF: myocardial blood flow; MPR: myocardial perfusion reserve; QoL: quality of life; RA: refractory angina; SPECT: single photon emission computed tomography; WMSI: wall motion score index.

## Data Availability

No new data were created or analyzed in this study. Data sharing is not applicable to this article.
